# Modelling multiple thresholds in meta-analysis of diagnostic test accuracy studies

**DOI:** 10.1186/s12874-016-0196-1

**Published:** 2016-08-12

**Authors:** Susanne Steinhauser, Martin Schumacher, Gerta Rücker

**Affiliations:** 1 Institute for Medical Biometry and Statistics, Faculty of Medicine and Medical Center – University of Freiburg, Freiburg, Stefan-Meier-Strasse 2679104 Germany; 2 Institute of Medical Statistics, Informatics and Epidemiology, University of Cologne, Cologne, Kerpener Str. 6250937 Germany

**Keywords:** Diagnostic accuracy study, Meta-analysis, Biomarker, Threshold, ROC curve

## Abstract

**Background:**

In meta-analyses of diagnostic test accuracy, routinely only one pair of sensitivity and specificity per study is used. However, for tests based on a biomarker or a questionnaire often more than one threshold and the corresponding values of true positives, true negatives, false positives and false negatives are known.

**Methods:**

We present a new meta-analysis approach using this additional information. It is based on the idea of estimating the distribution functions of the underlying biomarker or questionnaire within the non-diseased and diseased individuals. Assuming a normal or logistic distribution, we estimate the distribution parameters in both groups applying a linear mixed effects model to the transformed data. The model accounts for across-study heterogeneity and dependence of sensitivity and specificity. In addition, a simulation study is presented.

**Results:**

We obtain a summary receiver operating characteristic (SROC) curve as well as the pooled sensitivity and specificity at every specific threshold. Furthermore, the determination of an optimal threshold across studies is possible through maximization of the Youden index. We demonstrate our approach using two meta-analyses of B type natriuretic peptide in heart failure and procalcitonin as a marker for sepsis.

**Conclusions:**

Our approach uses all the available information and results in an estimation not only of the performance of the biomarker but also of the threshold at which the optimal performance can be expected.

**Electronic supplementary material:**

The online version of this article (doi:10.1186/s12874-016-0196-1) contains supplementary material, which is available to authorized users.

## Background

Systematic reviews of diagnostic test accuracy (DTA) studies give an overview of the performance of a diagnostic test, e.g. based on a biomarker or a questionnaire. Meta-analysis of DTA studies is traditionally based on one pair of sensitivity and specificity (Se, Sp) per study. Thus each study contributes a two by two table, containing the numbers of true positives (TP), false positives (FP), true negatives (TN) and false negatives (FN). The aims are twofold: On the one hand, one wants to estimate the pooled sensitivity and specificity with confidence regions. The assumption here is that all studies used similar thresholds for the biomarker underlying the test. On the other hand, if varying thresholds were used in the studies, a summary receiver operating characteristic (SROC) curve is estimated to describe the change in sensitivity and specificity while varying the threshold [1].

There are a number of published systematic reviews where several studies reported more than one threshold and the corresponding values of sensitivity and specificity, and also the thresholds were provided (see for example [2–6]). When using the standard bivariate meta-analysis model, however, one threshold value per study must be selected, and the additional information is ignored. In many cases the selected threshold is optimal with respect to the Youden index, which may lead to a too optimistic evaluation of the biomarker [5, 7, 8]. Thus, it is advantageous to use all the available data. As Leeflang et al. noted, ‘At present, the routinely used models for DTA meta-analysis utilise data on a single sensitivity and specificity pair for each study. Hence, current models do not fully utilise all of the available data. Some progress has been made in this area [9], but more general and robust methods are required’ [10].

Our motivation to work on a new approach is also due to our experience that clinicians often ask at which threshold of the biomarker the diagnostic test performs best. They expect meta-analysis to answer this question. Therefore methods to determine such an optimal threshold across all studies are urgently awaited. We note that methods focussing on ROC curves, ignoring the underlying biomarker, are not appropriate to answer this question.

There are already existing approaches which make use of more than one pair of sensitivity and specificity per study. An early approach was by Dukic and Gatsonis who used ordinal regression accounting for varying number of thresholds [11], including a Bayesian hierarchical approach. The multivariate random effects approach proposed by Hamza et al. [9] is a generalization of the standard bivariate model, which assumes an equal number of thresholds per study. Putter et al. [12], showing a case with common thresholds, used methods from survival analysis, modelling the marker distributions using a Poisson correlated gamma frailty model. Martínez-Camblor [13] suggested a non-parametric approach directly averaging the within-study ROC curves. Riley and coauthors also proposed two multivariate regression models, both in a diagnostic and in a prognostic context. One of these (option (ii) in [14], subsection 3.2 in [15]) models a functional relationship and is related to our approach. The problem of incomplete reporting of thresholds is discussed in [8].

We present a new approach for meta-analyses of DTA studies adapted to this more extensive type of data. It leads to pooled estimates of sensitivity and specificity as well as to an SROC curve. Furthermore, an optimal threshold across studies can be determined. The fundamental idea is to estimate the distribution functions of the biomarker within the diseased and non-diseased individuals using a linear mixed effects model.

The article is structured as follows. In the next section, after reviewing the standard models, we present our new approach, including determination of an SROC curve and finding an optimal threshold. In the results section we describe the results of a simulation study and apply our approach to two meta-analyses from the literature. After the discussion section we end with conclusions.

## Methods

### Standard models for meta-analysis of DTA studies

The hierarchical model was originally presented in a Bayesian framework [16, 17]. The parameters in the hierarchical model are *Θ* and *Λ*, together with their variances, and a shape parameter *β* which is related to the variance ratio of the two distributions. *Θ* represents the average logit probability of a positive test result (‘positivity’ [16, 18]) across all studies and groups of patients. The *θ*s for the studies are drawn from a normal distribution with mean *Θ* and model differences in ‘positivity’ which are due to different thresholds across studies. *Λ* is the average difference of the expectations of the distributions on the logit scale, that is, a log diagnostic odds ratio, and models accuracy.

Another widely used approach for meta-analysis of DTA studies is the bivariate model [19, 20], a random effects model focussing on the joint normal distribution of the logit-transformed sensitivity and specificity. The bivariate model has two levels and aims to pool sensitivity and specificity. At the study level, the numbers TP and FP of individuals with a positive test result from study *s*, *s*=1,…,*m*, are assumed to be independent and to follow binomial distributions

$$\begin{array}{*{20}l} \text{TP}_{s} & \sim \text{Binomial}(n_{1s}, \text{Se}_{s}),\\ \text{FP}_{s} & \sim \text{Binomial}(n_{0s}, 1-\text{Sp}_{s}), \end{array} $$

where index *s* indicates study *s* and *n*_1*s*_ and *n*_0*s*_ are the number of diseased and non-diseased individuals in study *s*. Throughout this article diseased individuals will always be denoted by 1 and non-diseased by 0. At the between-study level, logit-transformed sensitivity and 1−specificity are assumed to follow a bivariate normal distribution:

$$\begin{array}{@{}rcl@{}} \left(\begin{array}{c} \text{logit}(\text{Se}_{s})\\ \text{logit}(1-\text{Sp}_{s}) \end{array}\right) \sim N\left(\begin{array}{c} \left(\begin{array}{c} \mu_{1}\\ \mu_{0} \end{array}\right), \left(\begin{array}{rr} {\tau_{1}^{2}} & \tau_{10} \\ \tau_{10} & {\tau_{0}^{2}} \end{array}\right) \end{array}\right) \end{array} $$

Thus the two-dimensional nature of the data is preserved and the variability between the studies is taken into account with random effects. It has been shown that in case of no covariates, the hierarchical model and the bivariate model are equivalent [18, 21].

These standard models are based on the assumption that each study in a meta-analysis contributes only one pair of sensitivity and specificity. This leads to the problem of a not uniquely defined SROC curve, as there are many different ways to define the straight line in logit space [21]. Furthermore, the SROC curve might be overestimated as most studies will report a kind of optimal pair of sensitivity and specificity [7]. If studies present more than one threshold, the meta-analyst needs to reduce the data and select a threshold. This procedure does not use the full information [10] and also may lead to bias. As the underlying threshold is ignored in the models, no optimal threshold can be determined.

### New parametric approach based on several thresholds per study

The novel approach we want to present is characterized by the estimation of the cumulative distribution functions of the biomarker the test is based on within the non-diseased and diseased individuals, respectively [22]. This approach is applicable if several studies of a meta-analysis report more than one threshold and the corresponding values of sensitivity and specificity. More specifically, for each threshold reported by a study to be included in the meta-analysis, we need the threshold and the numbers of TP, FP, TN and FN.

We consider a continuous biomarker that is observed in each individual of two groups, non-diseased and diseased. Given a fixed threshold of the biomarker, without loss of generality, a test result is defined as positive if the observed value exceeds the threshold. We focus on the probability of negative test results within the non-diseased individuals (specificity) and within the diseased (1−sensitivity). Specificity and 1−sensitivity are interpreted as functions of the threshold *x*: the specificities provide data points of the cumulative distribution function (cdf) of the biomarker for the non-diseased individuals, the 1−sensitivities provide data points of the cdf for the diseased individuals. We make some distributional assumption for the biomarker, for example, we may assume a normal or logistic distribution. In parentheses, we note that we could as well, equivalently, model the ‘survival’ functions instead of the cdfs, which would mean to focus at 1−specificity and sensitivity, like in the ROC curve.

For each study, an arbitrary number of thresholds (not necessarily equal across studies) and the numbers of TP, FP, FN and TN for each threshold are assumed to be known. With this data we aim to estimate the parameters of the distribution functions of the biomarker within the non-diseased and diseased, respectively.

Transforming sensitivity and specificity so that they are linear in the threshold enables us to use a linear model to fit the data. We chose an appropriate transformation, that is, a function *h*, for example, *h*=*Φ*^−1^ (normal model; *Φ*^−1^ denotes the inverse of the standard normal distribution) or *h*=logit (logistic distribution model). Let $(\mu _{0},{\sigma _{0}^{2}})$ be the mean and variance parameters of the biomarker distribution for the non-diseased individuals and $(\mu _{1},{\sigma _{1}^{2}})$ the parameters for the diseased. Let *x* be a threshold. We obtain the linear equations

1 $$\begin{array}{*{20}l} h\left(\text{Sp}(x)\right) &= \frac{x - \mu_{0}}{\sigma_{0}}, \end{array} $$

2 $$\begin{array}{*{20}l} h\left(1 -\text{Se}(x)\right) & = \frac{x - \mu_{1}}{\sigma_{1}}, \end{array} $$

where *h* is the transformation.

In the following, we want to fit the transformed data. To account for the clear hierarchical structure and the heterogeneity of the studies, we consider the studies as randomly chosen out of the overall study population and regress the data with a linear mixed effects model with study as grouping factor. We want to explain the transformed proportions of negative test results, with TN_*si*_/*n*_0*s*_ being the proportion of negative test results of the non-diseased of study *s,s*=1,…,*m* and the threshold indexed by *i,i*=1,…,*k*_*s*_, and FN_*si*_/*n*_1*s*_ the one of the diseased, in dependence of the thresholds *x*_*si*_. To obtain different location and dispersion parameters of the biomarker distributions within both groups, we estimate separate regression lines for the non-diseased and diseased, respectively. We consider a class of weighted linear mixed effects regression models, with fixed effects for group and threshold and their interaction and different random effects. The most general linear mixed model contains four fixed effects (*α*_0_,*α*_1_,*β*_0_,*β*_1_) and four random effects (*a*_0*s*_,*a*_1*s*_,*b*_0*s*_,*b*_1*s*_). The random effects are assumed to follow a multivariate normal distribution with mean zero and a completely general variance matrix. The model is given by

$${} \begin{aligned} &h\left(\!\frac{\text{TN}_{si}}{n_{0s}}\!\right)\,=\,\alpha_{0} + \!a_{0s}+ \!(\beta_{0} + \!b_{0s})x_{si} + e_{si}, (\textsc{model} *\text{DIDS}) \\ &h\left(\frac{\text{FN}_{si}}{n_{1s}}\right)=\alpha_{1} + a_{1s} + (\beta_{1} + b_{1s})x_{si} + f_{si},\\ &(a_{0s},a_{1s}, b_{0s},b_{1s}) \\ &\sim N\left(\!0, \left(\!\begin{array}{cccc} \tau_{0a}^{2} & ~~~\rho_{1}\tau_{0a}\tau_{1a} & ~~~\rho_{2}\tau_{0a}\tau_{0b} & ~~~\rho_{3}\tau_{0a}\tau_{1b} \\ \rho_{1}\tau_{0a}\tau_{1a} & \tau_{1a}^{2} &\rho_{4}\tau_{1a}\tau_{0b} & \rho_{5}\tau_{1a}\tau_{1b}\\ \rho_{2}\tau_{0a}\tau_{0b} & \rho_{4}\tau_{1a}\tau_{0b} & \tau_{0b}^{2} & \rho_{6}\tau_{0b}\tau_{1b}\\ \rho_{3}\tau_{0a}\tau_{1b} & \rho_{5}\tau_{1a}\tau_{1b} & \rho_{6}\tau_{0b}\tau_{1b} & \tau_{1b}^{2} \end{array}\right)\! \right)\!,\\ & e_{si}\sim N\left(0, \frac{\gamma^{2}}{w_{si}}\right),\\ &f_{si} \sim N\left(0,\frac{\gamma^{2}}{v_{si}}\right),\qquad s=1,\dots,m,~ i=1,\dots,k_{s}, \end{aligned} $$

where *α*_0_ and *α*_1_ are the fixed intercepts and *β*_0_ and *β*_1_ the fixed slopes for the non-diseased and diseased, respectively. The explanatory variable *x*_*si*_ is the *i*^th^ threshold of study *s*. The independent error terms of the non-diseased are denoted with *e*_*si*_, the ones of the diseased with *f*_*si*_ for the *i*^th^ threshold of study *s*. They are both mean zero normally distributed with variances *γ*^2^/*w*_*si*_ and *γ*^2^/*v*_*si*_, respectively, where *γ* is an unknown scale parameter (which is estimated) and *w*_*si*_ and *v*_*si*_ are given prior weights. As prior weights we propose either sample size or inverse variance scaled to mean one.

The random intercepts of non-diseased and diseased individuals are denoted *a*_0*s*_ and *a*_1*s*_, respectively, and the random slopes of non-diseased and diseased individuals *b*_0*s*_ and *b*_1*s*_, respectively. Whereas diseased and non-diseased individuals within the same study are not correlated, the across-study correlation must be modeled (parameters *ρ*_1_,…,*ρ*_6_). The residual errors *e*_*si*_ and *f*_*si*_ are independent of the random intercepts and slopes.

The model described above is named *DIDS, **D**ifferent random **I**ntercept and **D**ifferent random **S**lope. As the total number of parameters to estimate is quite large, a lot of data is needed to enable use of model *DIDS for estimation. To reduce the model we want to either consider fewer random effects or equalize random effects within the non-diseased and diseased but will not restrict the correlation matrix (see Table 1). For all of these models there is a simplified variant which forces the fixed effect slopes for the diseased and non-diseased individuals into being equal, i.e., *β*_0_=*β*_1_. To distinguish them from the general models, we mark the general models with ‘*’. Thus, in total we obtain 16 different models.

**Table 1:** Linear mixed effects models listed according to their random effects structure

Model	Specification
DIDS	Different random intercepts and different random slopes
CIDS	Common random intercept and different random slopes,
	*a*_0*s*_=*a*_1*s*_=*a*_*s*_
DICS	Different random intercepts and common random slope,
	*b*_0*s*_=*b*_1*s*_=*b*_*s*_
CICS	Common random intercept and common slope,
	*a*_0*s*_=*a*_1*s*_=*a*_*s*_, *b*_0*s*_=*b*_1*s*_=*b*_*s*_
DS	Different random slopes,
	*a*_0*s*_=*a*_1*s*_=0
CS	Common random slope,
	*a*_0*s*_=*a*_1*s*_=0, *b*_0*s*_=*b*_1*s*_=*b*_*s*_
DI	Different random intercepts,
	*b*_0*s*_=*b*_1*s*_=0
CI	Common random intercept,
	*a*_0*s*_=*a*_1*s*_=*a*_*s*_, *b*_0*s*_=*b*_1*s*_=0

To choose between models, we first decided on using either the simplified models or the general ones. Then, we applied the REML (restricted maximum likelihood) criterion [23, 24], which selects the most suitable model of a range of models with same fixed effects and differing random effects. Finally, the model with the smallest REML criterion was selected.

Back-transforming the model equation using *h*^−1^ (e.g., *Φ* in the normal case or logit^−1^ if a logistic distribution is assumed) provides the model-based distribution functions of the biomarker for non-diseased and diseased individuals. For example, in the normal case, the estimated distribution parameters $\hat \mu _{j}, \hat \sigma _{j}$, *j*=0,1, are provided by the fixed effects parameters (see Eqs. (1), (2)) by

$$\begin{array}{*{20}l} \hat\mu_{j} = - \frac{\alpha_{j}}{\beta_{j}}, \quad \hat\sigma_{j} = \frac{1}{\beta_{j}} \quad (j = 0,1). \end{array} $$

Thus, it is necessary that the *β*_*j*_ (*j*=0,1) are positive to obtain positive dispersions. That means specificity and 1−sensitivity, i.e. the probabilities of having a negative test result, should increase with increasing thresholds within both groups over all studies. If a logistic distribution assumption is used, the $\hat \sigma _{j}~(j = 0,1)$ have to be multiplied with $\pi /\sqrt {3}$ to obtain standard deviations. As we can see, if one fixes *β*_0_=*β*_1_ in the linear regression models, one assumes that the distributions of the biomarker of non-diseased and diseased individuals have equal variances.

For estimation we used the lmer() function in R [25] with REML estimation and inverse variance weights scaled to mean one [26]. To avoid problems with zero values, we added a continuity correction of 0.5 to the numbers TN_*si*_, TP_*si*_, FN_*si*_ and FP_*si*_. In case of the logit transformation, the Delta method (with continuity correction) leads to the variance estimates (TN_*si*_+0.5)^−1^+(FP_*si*_+0.5)^−1^ (disease-free) and (TP_*si*_+0.5)^−1^+(FN_*si*_+0.5)^−1^ (diseased) and the corresponding inverse variance weights. For the probit transformation *h*=*Φ*^−1^, the Delta method leads to analogous weights, see the R code provided in Additional file 1.

To demonstrate our models on examples, we used only models of the general form, i.e. where the fixed slopes of non-diseased and diseased individuals may differ, because these models performed better in the simulation study (models indicated by ‘*’). To choose one model of this range, we selected the one with the smallest REML criterion. We used a weighting parameter *λ*_*w*_ of 0.5, meaning that sensitivity and specificity were equally weighted.

### SROC curve and optimal threshold

Once the model parameters are estimated, the underlying distribution functions are determined. From these, one can read off the pooled sensitivity and specificity values at every threshold and also specify confidence regions. A SROC curve and an optimal threshold are also derived.

**Sensitivity, specificity, confidence regions** We derived confidence intervals as follows. From the given lmer() object, we extracted the estimates (hats omitted) of *α*_0_,*α*_1_,*β*_0_,*β*_1_,Var(*α*_0_),Var(*α*_1_),Var(*β*_0_),Var(*β*_1_),Cov(*α*_0_,*β*_0_),Cov(*α*_1_,*β*_1_).

Given a threshold *x*, specificity and sensitivity were obtained by back-transforming the linear regression estimates using *h*^−1^:

$$\begin{array}{*{20}l} \text{Sp}(x) &= h^{-1}(\alpha_{0} + \beta_{0}x)\\ \text{Se}(x) &= 1 - h^{-1}(\alpha_{1} + \beta_{1}x) \end{array} $$

The sampling variances for the transformed specificities and sensitivities, conditional on the threshold *x*, are

$$\begin{array}{@{}rcl@{}} \text{Var}(\alpha_{0} + \beta_{0}x) &= \text{Var}(\alpha_{0}) + x^{2} \ \text{Var}(\beta_{0}) + 2x \ \text{Cov}(\alpha_{0},\beta_{0})\\ \text{Var}(\alpha_{1} + \beta_{1}x) &= \text{Var}(\alpha_{1}) + x^{2} \ \text{Var}(\beta_{1}) + 2x \ \text{Cov}(\alpha_{1},\beta_{1}) \end{array} $$

Confidence bands were obtained by adding/subtracting the standard errors times the normal quantile *z*_0.975_ to the transformed estimates and back-transforming the confidence limits using *h*^−1^.

**SROC curve** The SROC curve naturally follows from the distributions by

$$\begin{array}{*{20}l} ROC(t)=1-F_{\mu_{1},\sigma_{1}}\left(F^{-1}_{\mu_{0},\sigma_{0}}\left(1-t\right)\right), \quad 0\leq t\leq 1, \end{array} $$

where *F*_*μ*,*σ*_ is the distribution function with location and scaling parameters *μ* and *σ*, e.g., *Φ*_*μ*,*σ*_ under normal assumption with mean *μ* and standard deviation *σ* [1].

**Youden index** The weighted Youden index *Y*_*w*_ for a threshold *x* is defined by

$$\begin{array}{*{20}l} Y_{w}(x) = 2 \ \left(\lambda_{w}\cdot \text{Se}(x) + (1-\lambda_{w}) \cdot \text{Sp}(x)\right) - 1, \end{array} $$

where *λ*_*w*_∈[0,1] is a weighting parameter [7]. To equally weight sensitivity and specificity a *λ*_*w*_ of 0.5 is chosen. To emphasize sensitivity, a higher value of *λ*_*w*_ and to emphasize specificity, a lower value is chosen. We can write the estimated weighted Youden index $\hat {Y}_{w}$ for a threshold *x* as

$$\begin{array}{@{}rcl@{}} \hat{Y}_{w}(x) &=& \lambda_{w} \left(1 - 2 h^{-1} \left(\frac{x - \hat\mu_{1}}{\hat\sigma_{1}}\right)\right)\\ &&+ (1-\lambda_{w}) \left(2 h^{-1} \left(\frac{x - \hat\mu_{0}}{\hat\sigma_{0}}\right) - 1 \right). \end{array} $$

The optimal threshold *x*_0_ is defined as the threshold which maximises the Youden index *Y*_*w*_(*x*). Under normal assumption, it can be estimated for $\hat \sigma _{0} \neq \hat \sigma _{1}$ by setting

$${} {\begin{aligned} \hat x_{0} &= \frac{\hat\mu_{0}{\hat\sigma_{1}^{2}} - \hat\mu_{1}{\hat\sigma_{0}^{2}}}{{\hat\sigma_{1}^{2}} -{\hat\sigma_{0}^{2}}}\\ &\quad+ \frac{\sqrt{{\hat\sigma_{0}^{2}}{\hat\sigma_{1}^{2}}\left(2({\hat\sigma_{1}^{2}} - {\hat\sigma_{0}^{2}})\left(\log \frac{\hat\sigma_{1}}{\hat\sigma_{0}} - \text{logit}(\lambda_{w})\right) + (\hat\mu_{1} - \hat\mu_{0})^{2}\right)}}{{\hat\sigma_{1}^{2}} - {\hat\sigma_{0}^{2}}} \end{aligned}} $$

(see [27]). For $\hat \sigma _{0} = \hat \sigma _{1}=:\hat \sigma $, $\hat x_{0}$ is given by

$$\begin{array}{*{20}l} \hat x_{0}=\frac{\hat\sigma^{2}\text{logit}(\lambda_{w}) + \frac{1}{2}\left({\hat\mu_{0}^{2}}-{\hat\mu_{1}^{2}}\right)}{\hat\mu_{0}-\hat\mu_{1}}. \end{array} $$

For the logistic distribution assumption of the biomarker, no analytical solution of the maximization problem of the Youden index has been found. Thus we implemented a fixed point iteration to compute the optimal threshold. For a discrete ordinal scale, the maximum can be found by maximizing the Youden index on the finite set of possible thresholds. For the normal distribution assumption, we also derived a confidence interval for the optimal threshold using the delta method which is implemented in our R code, see Additional file 1 (for details of the derivation, see (*§*3.3.6.3 [22])).

### Simulation study

To evaluate the performance of our method, we conducted a simulation study. We aimed to investigate how precisely the new approach can estimate the parameters of the true distributions of diseased and non-diseased individuals. Furthermore, we examined if the model is a suitable approach to estimate the pooled sensitivity and specificity and the optimal threshold in a meta-analysis. Therefore we considered 384 scenarios with 1000 runs each. Data was simulated mimicking roughly the example data. The values were drawn from the specified distributions or sets.

- Number of studies: 10, 20, 30
- True overall normal distributions of the biomarker:
  - Mean: 0/2.5 [non-diseased/diseased]
  - Standard deviation: 1.5/1.5 (‘same’), 1/2 (‘different’) [non-diseased/diseased]
- Random noise:
  To obtain study-specific distributions, random noise was added to the true overall distributions. The extent of the random noise was determined by a visual comparison with the examples.
  - To mean: *N*(0,*τ*^2^), *τ*= 0 (‘no heterogeneity’), 0.5 (‘moderate heterogeneity’), 1 (‘large heterogeneity’) or 1.5 (‘huge heterogeneity’),
    symmetrically truncated so that the mean of the study-specific distribution of the diseased individuals was greater than that of the non-diseased
  - To standard deviation: *N*(0,*τ*^2^), *τ*= 0, 0.3, 0.4 or 0.5 likewise, symmetrically truncated in order to guarantee non-negative study-specific standard deviations
- Total number of individuals per study:
  Lognormal(5, 1)
- Proportion of diseased individuals: *N*(0.5,0.04) truncated to the interval (0.2, 0.8)
- Number of thresholds per study: Pois(*λ*=1.3 or 2), rejecting zeros, or fixed to 5
- Values of thresholds: spaced equidistantly between the 40 % quantile of the study-specific distribution of the non-diseased individuals and the 60 % quantile of the study-specific distribution of the diseased individuals
- True sensitivity and specificity: Once the distributions were fixed, the true sensitivity and the true specificity were derived as the areas under the respective curves to both sides of the threshold. Sensitivity and specificity were equally weighted.
- True optimal threshold: The point where the densities cut was defined as the true optimal threshold. That is, we defined the optimal threshold as the point where the Youden index was maximized, weighting sensitivity and specificity equally.
- Models: CI, DS, CICS, CIDS, *CI, *DS, *CICS, *CIDS

We did not include the most complex models DIDS and *DIDS because there was mostly insufficient data. For the computational implementation of the linear random effect models we used the lmer() function of the R package lme4_1.1-7 with REML estimation. For weighting of the studies we used inverse variance weights scaled to mean one.

We investigated bias, mean squared error (MSE) and coverage of the distribution parameters *μ*_0_,*μ*_1_,*σ*_0_ and *σ*_1_ and of sensitivity and specificity at three points: at 0, at the true optimal threshold and at 2.5. Furthermore, we investigated bias and MSE for the optimal threshold. In addition, we documented how often error messages occurred, particularly how often a negative slope was observed (making model estimation impossible), and the percentage of runs where a warning message signaled that convergence could not be achieved.

## Results

### Results of the simulation study

**Fig. 1:**
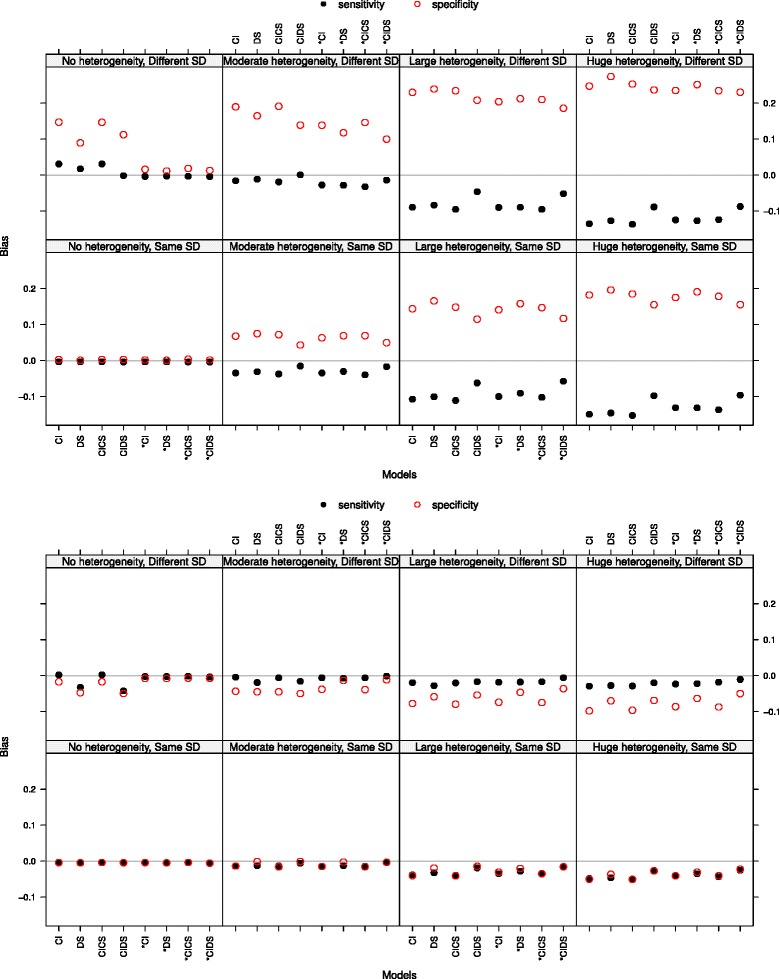
Bias of sensitivity and specificity at threshold 0 (*top panel*) and at the true optimal threshold (*bottom panel*). The Poisson distribution was chosen with *λ*=1.3 for the number of thresholds. The four plots at the bottom show the case of same standard deviations (SD), the top four plots the case of different standard deviations. The heterogeneity of the studies increases from *left* to *right*

**Sensitivity and specificity: bias and mean squared error** The bias of sensitivity and specificity increased with increasing heterogeneity (see Fig. 1 at threshold 0 and at the true optimal threshold, both with a Poisson distribution parameter *λ*=1.3 for the number of thresholds). At the true optimal threshold the bias was markedly smaller than at the points 0 and 2.5, not overpassing an absolute value of 0.12 and almost always underestimating the values. At threshold 0 sensitivity was underestimated and specificity was overestimated, at threshold 2.5 this held vice versa (not shown). Thus, small values of sensitivity and specificity were overestimated and large ones underestimated. In the case of no heterogeneity there was nearly no bias for data with same standard deviations (SD), whereas for different SD (upper rows of the plots in Fig. 1) the models assuming same SD (the ones without ‘*’) led to bias. An explanation could be that the data is quite perfect, as there is no heterogeneity, but the slopes of the two straight lines to be estimated are forced to be equal and thus all parameters suffer. This phenomenon vanished with more heterogeneity. The bias in the case of different SD was slightly larger than in the case of same SD at the point 0 and the true optimal threshold and slightly smaller at point 2.5. The bias decreased with an increasing number of thresholds at points 0 and 2.5. At the true optimal threshold there was no impact. With more thresholds we observe a zigzag pattern, with the highest bias resulting from model DS and *DS and the lowest from CIDS and *CIDS (not shown). The mean squared error behaved similarly to the bias and thus will not be discussed.

**Fig. 2:**
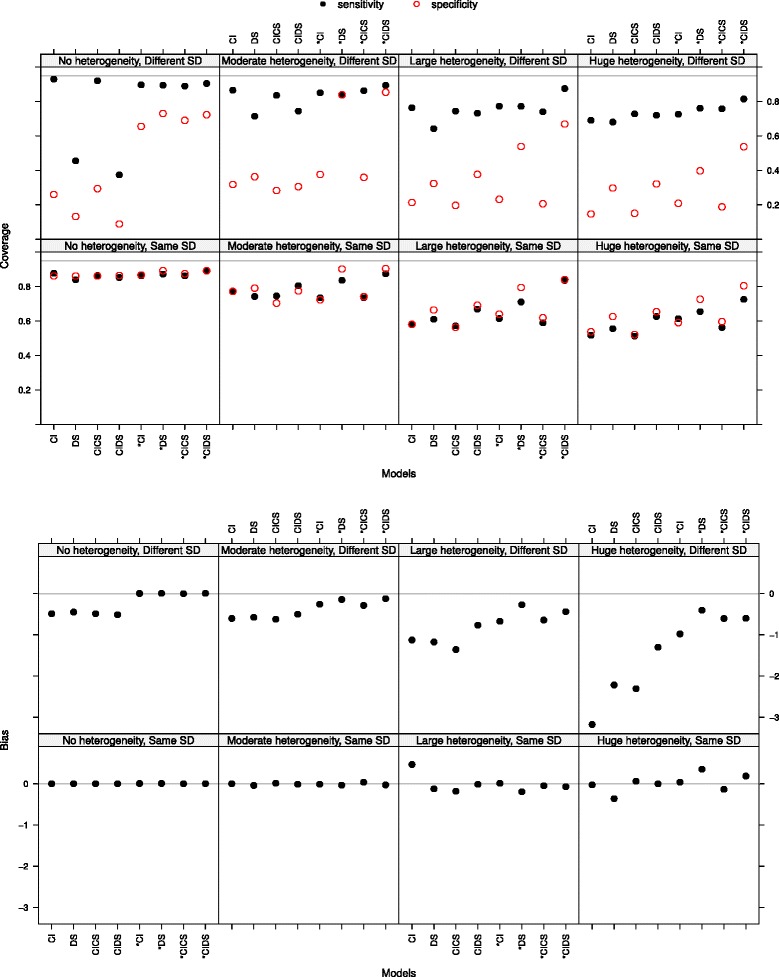
Coverage of sensitivity and specificity at the true optimal threshold (*top panel*). Bias of the optimal threshold (*bottom panel*). The Poisson distribution was chosen with *λ*=1.3 for the number of thresholds. The four plots at the bottom show the case of same standard deviations (SD), the top four plots the case of different standard deviations. The heterogeneity of the studies increases from *left* to *right*

**Sensitivity and specificity: coverage of 95 % confidence intervals** The coverage of sensitivity and specificity was decreasing with increasing heterogeneity, being smaller in the case of different SD (see the top panel in Fig. 2 at the true optimal threshold with a Poisson distribution parameter *λ*=1.3 for the number of thresholds). In case of no heterogeneity and different SD, models which force equal fixed slopes led to smaller coverage. This may be explained by a small confidence interval due to no heterogeneity and existing bias. The coverage did not improve with an increasing number of thresholds per study.

**Distribution parameters** The results of the estimation of the distribution parameters will not be discussed in detail, as the results were very similar to the ones of sensitivity and specificity and it is the primary goal to estimate correct sensitivity and specificity. There were outliers of bias of the distribution parameters reaching values up to 100 for few thresholds per study, but generally the bias decreased markedly with increasing number of thresholds.

**Optimal threshold** In the meta-analysis an overall optimal threshold was estimated. The bias of this optimal threshold was small but slightly increasing with increasing heterogeneity (see the bottom panel of Fig. 2 with a Poisson distribution parameter *λ*=1.3 for the number of thresholds). It was smaller in the case of same standard deviations (plots at the bottom) than in the case of different standard deviations. There the bias of models forcing equal slopes was markedly higher than the one of models allowing for different slopes. With increasing number of thresholds per study the bias was decreasing (not shown). The MSE behaved similar to the bias and thus will not be discussed.

**Fig. 3:**
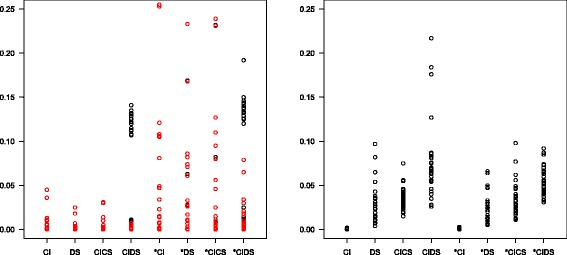
Percentage of error messages (*left panel*) and warnings (*right panel*) in simulation scenarios with a varying number of thresholds. *Red circles* indicate cases with negative regression slope

**Fig. 4:**
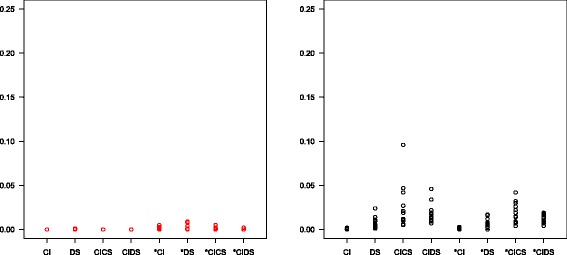
Percentage of error messages (*left panel*) and warnings (*right panel*) in simulation scenarios with the number of thresholds fixed to five. *Red circles* indicate cases with negative regression slope

**Problems with negative slope and non-convergence** Figure 3 shows the proportion of errors (left) and warnings (right) in 1000 simulation runs with varying number of thresholds, distribution parameters and random noise, separated by model. The boxplots and black circles in the left figure represent the total number of error messages, the red circles the number of error messages due to a negative regression slope. This occurred more frequently with the ‘*’ models that have to estimate two different slopes (up to a quarter of runs), particularly if the number of thresholds was small and/or heterogeneity was large. Another possible reason for error was that the threshold iteration did not converge. The right panel shows the proportion of warnings signaling that convergence could not be achieved. This was more frequent for more complex models. Figure 4 provides the corresponding information for 1000 simulation runs with number of thresholds fixed to five. Further simulations showed that all kinds of errors and warnings were much less frequent or even completely vanished if there was more threshold information and/or if there were many studies in a meta-analysis (not shown).

### Examples

To illustrate our approach we applied it to two data sets of published meta-analyses, both with a continuous marker. We will obtain pooled sensitivity and specificity, an optimal threshold and a SROC curve. For both examples we chose the logit transformation for comparing the result with those in the original publications.

#### Example 1: Diagnostic accuracy of B type natriuretic peptides in heart failure

In a recent meta-analysis Roberts et al. investigated the diagnostic accuracy of, among others, B type natriuretic peptide in heart failure and found 26 studies, where several were reporting more than one threshold ([2], Fig. 1). To use the standard bivariate model, they grouped the data according to recommended thresholds and performed two meta-analyses (100 ng/L and 100–500 ng/L). For thresholds ≥ 500 ng/L, Roberts et al. did no meta-analysis because there were only four studies that showed much heterogeneity.

**Fig. 5:**
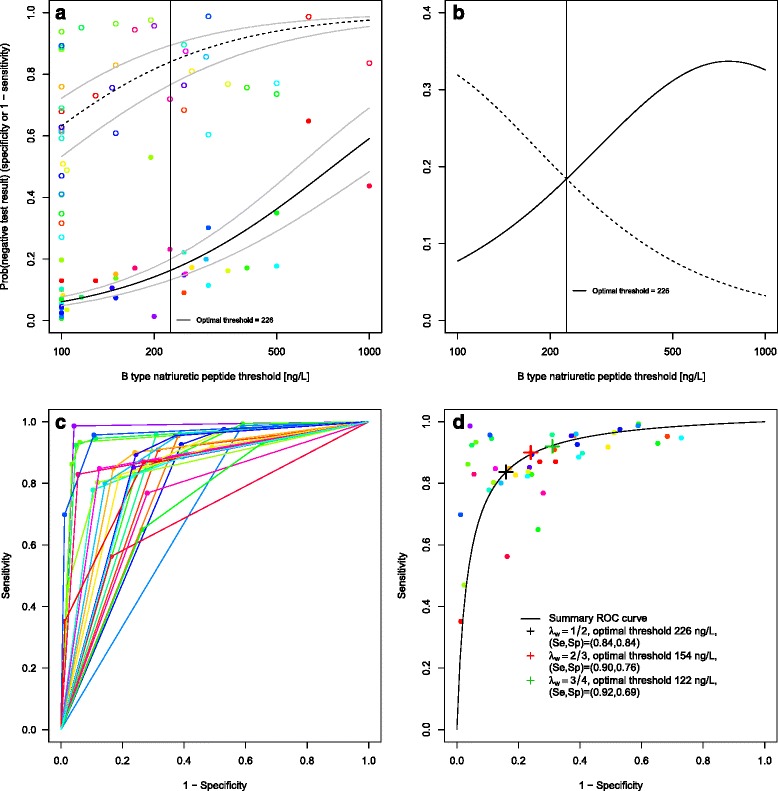
B type natriuretic peptide data. **a** Estimated distribution functions for the non-diseased (*open circles, dashed line*) and the diseased individuals (*filled circles, solid line*). The grey lines mark the confidence regions and different studies are marked in different colours. The optimal threshold, derived from a maximization of the Youden index, is depicted as a solid vertical line. **b** Estimated densities and their point of intersection. Non-diseased (*dashed line*), diseased individuals (*solid line*). **c** Study-specific ROC curves. **d** Estimated SROC curve with the optimal thresholds for different weightings of sensitivity and specificity marked as crosses in black, red and green. Different studies are marked in different colours

However, meta-analyses of the same studies based on different thresholds are correlated. We thus performed one meta-analysis including all the data of the B type natriuretic peptide by Roberts et al. [2]. We log-transformed the threshold data and then used a logistic distribution assumption, in analogy to the logit transformation in the bivariate model. Together, this means a log-logistic distribution assumption for the biomarker. REML was minimised by model *DICS. The results of our approach are seen in Table 2. At the optimal threshold of 226.0 ng/L, sensitivity was 0.84 with a 95 % confidence interval of [0.80, 0.87] and specificity was also 0.84 [0.77, 0.89]. Having estimated the biomarker distributions of the non-diseased and diseased, we may read off values of diagnostic accuracy for arbitrary thresholds. For 100 ng/L, the point estimates and confidence intervals of both methods agree nearly perfectly. Also the results by Roberts et al. for 100–500 ng/L agree well with our own for the optimal threshold, 226 ng/L. Our analysis gives model-based estimates also for the region 500–1000 ng/L, but they differ from those of each of the single studies given by Roberts et al. [2]. As most of the studies were carried out in the emergency department, it seems likely to emphasize sensitivity. This could be achieved in choosing *λ*_*w*_ larger than 0.5, such as *λ*_*w*_=2/3 or 3/4. This leads to an optimal threshold of 154.4 ng/L with a sensitivity of 0.90 [0.87, 0.92] and specificity of 0.76 [0.67, 0.83] for *λ*_*w*_=2/3 and an optimal threshold of 122.0 ng/L with a sensitivity of 0.92 [0.90, 0.94] and a specificity of 0.69 [0.60, 0.78] for *λ*_*w*_=3/4. Figure 5a shows the model-based cumulative log-logistic marker distributions for non-diseased and diseased individuals, Fig. 5b the estimated densities. Figure 5c shows the study-specific ROC curves. Figure 5d illustrates the SROC curve based on this model with the three different optimal thresholds for different choices of *λ*_*w*_ indicated. The R code (Additional files 1 and 2) and data sets (Additional files 3 and 4) to apply the method can be found as supporting information to this article.

**Table 2:** Sensitivity and specificity for selected thresholds, based on model *DICS and compared to results by Roberts et al. [2] (for thresholds greater or equal to 500 ng/L, Roberts et al. performed no meta-analysis)

	New model		Roberts et al. [2]	
	point estimate [95 % confidence interval]		point estimate [95 % confidence interval]	
Threshold [ng/L]	Sensitivity	Specificity	Sensitivity	Specificity
100	0.94 [0.92, 0.95]	0.63 [0.53, 0.72]	0.95 [0.93, 0.96]	0.63 [0.52, 0.73]
226	0.84 [0.80, 0.87]	0.84 [0.77, 0.90]	0.85 [0.81, 0.88]	0.86 [0.79, 0.91]
500	0.64 [0.56, 0.71]	0.94 [0.90, 0.97]	-	-
1000	0.41 [0.31, 0.52]	0.98 [0.96, 0.99]	-	-

#### Example 2: Procalcitonin as a diagnostic marker for sepsis

Wacker et al. [5] published a systematic review on the diagnostic accuracy of procalcitonin as a diagnostic marker for sepsis. Though 11 of the 31 primary studies had reported sensitivity and specificity at different (up to five) thresholds, the authors chose one pair of sensitivity and specificity per study for their meta-analysis using the bivariate model. They obtained a pooled sensitivity of 0.77 [0.72; 0.81] and a specificity of 0.79 [0.74; 0.84].

We extracted data for additional thresholds from the primary studies and found 54 data points in total for 26 different values of the threshold.

**Fig. 6:**
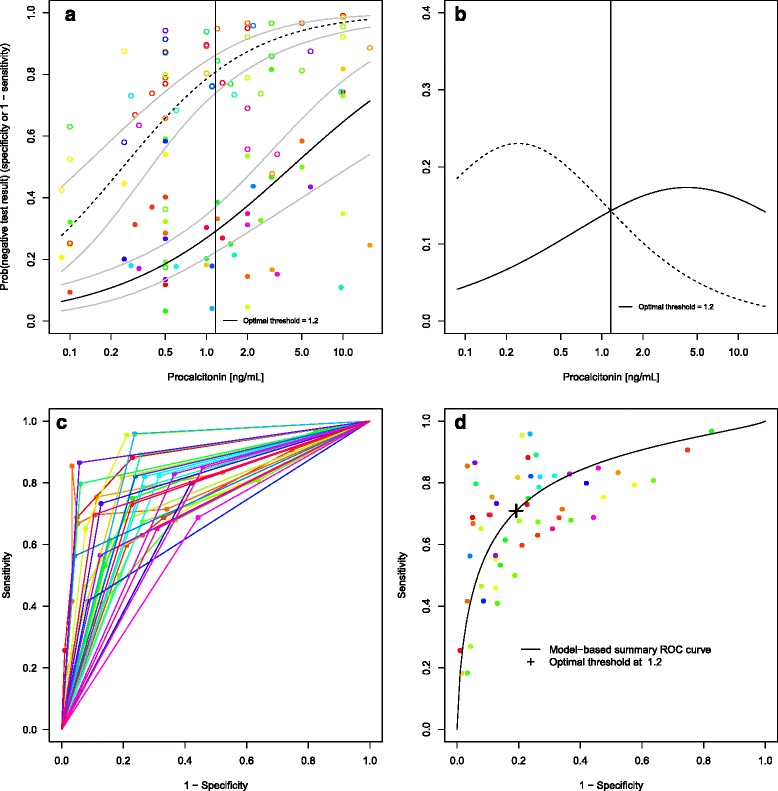
Procalcitonin data. **a** Estimated distribution functions for the non-diseased (*open circles, dashed line*) and the diseased individuals (*filled circles, solid line*). The grey lines mark the confidence regions and different studies are marked in different colours. The optimal threshold, derived from a maximization of the Youden index, is depicted as a *solid vertical line*. **b** Estimated densities and their point of intersection. Non-diseased (*dashed line*), diseased individuals (*solid line*). **c** Study-specific ROC curves. **d** Estimated SROC curve with the optimal threshold for equal weighting of sensitivity and specificity. Different studies are marked in different colours

Again, model *DICS minimized the REML criterion. This resulted in an estimated optimal threshold of 1.2 ng/mL with a sensitivity of 0.71 [0.63; 0.78] and a specificity of 0.81 [0.74; 0.86]. The results are shown in Fig. 6 which is structured like Fig. 5. Whereas the estimate of specificity is similar to that given in [5], the sensitivity estimate is more conservative. A possible reason is overoptimism due to selection of optimal thresholds when using the bivariate model [28].

## Discussion

We have described and evaluated a new approach for meta-analysis of diagnostic test accuracy studies, where several studies report more than one threshold and the corresponding values of sensitivity and specificity. The approach uses a common parametric assumption (normal or logistic) for the distribution of a continuous biomarker. The idea is to estimate the distribution functions of the biomarker, one distribution function within the non-diseased and one within the diseased study population. This is achieved by the use of a mixed effects model with study as random factor.

We applied our approach to a number of examples with both continuous biomarkers and ordinal questionnaires. Here we report results for two continuous biomarkers. In both examples we found large heterogeneity between the studies. Nevertheless, our approach led to convincing results, as the distribution functions and the pooled sensitivity and specificity with their confidence intervals seemed reasonable and were similar to already published results.

Our new approach for meta-analysis of DTA studies has its strengths and limitations.

**Strengths** Our approach uses multiple pairs of sensitivity and specificity and their corresponding thresholds per study. In comparison with traditional approaches, this has several advantages: we use all the given information and do not need to select one pair of sensitivity and specificity per study. After assuming a distribution type, we do not need additional assumptions for the SROC curve. In contrast to the alternative approaches of Hamza et al. [9] and Putter et al. [12], our approach can deal with a varying number of thresholds per study. We found varying numbers of thresholds in most of the systematic reviews providing multiple thresholds at all.

The models are based on a parametric assumption. The assumption of a normal or logistic distributed biomarker with different parameters for the non-diseased and diseased individuals is very common [1]. It seemed a natural idea to estimate underlying distributions. Directly and without further assumptions, we obtain all desired quantities: sensitivity and specificity, the SROC curve and the Youden index and the optimal threshold. By using a mixed effects model we acknowledge the diversity of the studies, while the data of each study has in principle the same structure. By admitting correlated random effects, we respect the bivariate character of the study data.

The logit and the probit transformation often provided similar results. By log-transforming the biomarker values, we can also handle skewed distributions (log-logistic or lognormal). In fact, each cumulative distribution function *F* can be transformed into a linear model by using the transformation *h*=*F*^−1^. In this way our basic idea can be extended to other distributions.

Standard approaches such as the bivariate model, and also the approach by Martínez-Camblor [13], are based solely on knowledge of pairs of sensitivity and specificity or ROC curves, without making use of threshold information. Often this information is missing in the primary studies. However, we found a number of reviews where this information was present or could be extracted from the primary studies in hindsight, and our approach establishes a link between threshold information and the ROC curve, and we may determine an optimal threshold among all studies. This is important information for clinicians. In the clinical routine it is not only of interest to know which is the best biomarker for a specific illness, but also at which threshold an optimal discrimination between non-diseased and diseased individuals can be achieved. The knowledge of a summary ROC curve alone does not allow inference on the biomarker.

Whereas most physiological biomarkers can be seen as continuous, questionnaires or psychological scales often take only integer values and therefore are ordinally scaled. However, in practice, they are often analyzed as continuous, also in meta-analyses [29]. Our approach could probably be used for psychological scales as well, but we did not systematically investigate this.

**Limitations** Our model, like the standard bivariate model, is a two-stage approach, based on the estimated transformed study-specific sensitivities and specificities and using inverse variance weights, however ignoring the uncertainty of their variances at the study level. It is thus a linear mixed model, not a generalized linear mixed model. A problem related to this is the necessity to use continuity correction, at least in case of zeros in the two by two tables which has been criticized [30].

The approach differs from others in that we did not use a binomial model for modeling sensitivity and specificity at the study level. This would have led to two binomial parameters per threshold with additional requirements of monotonicity and correlation ([14], option (i)). We think it is more natural to look at the distributions and refer to an analogous situation in survival analysis, where it is standard to consider a time-to-event variable, instead of jointly modeling binary outcomes such as, say, ‘one year mortality’, ‘two years mortality’ and ‘five years mortality’.

Some care has to be taken concerning the concept of an optimal threshold across studies. This is only reasonable if a biomarker value has the same meaning in all studies and does not differ because of laboratory conditions. If the thresholds are very heterogeneous, this has to be doubted. Of course the question arises as well in how far it is reasonable to pool sensitivity and specificity if the studies are very inhomogeneous.

A weak point of this method is the possibility of estimating decreasing proportions of negative test results with an increasing threshold. Whereas this is impossible within a study, it may happen if one combines data of several studies. Thus, if the heterogeneity between studies is huge and the number of thresholds is low, a valid regression slope cannot be assured and we do not recommend our method for such data. The problem becomes less relevant if there is sufficient threshold information.

If there are not enough data points reported from the studies, some of the linear mixed effects models may not be applicable as the number of parameters to be estimated might be too big. For some models and data sets, cases of numerical instability occurred. Besides, the fixed point iteration of the optimal threshold in case of a logistic distribution assumption did not converge in some few cases.

Model selection remains a challenge. We investigated several approaches, including the Akaike information criterion (AIC) and the conditional AIC (cAIC) criterion that allows comparing mixed models with different fixed effects [23, 31]. However, we encountered problems with cAIC, as the ordering of models surprisingly depended on how the weights were scaled, which seemed unplausible. We thus decided to apply the REML criterion [23].

Our R code offers a broad range of models, and users may decide which model or which selection criteria they want to use.

Further potential extensions of the method are the derivation of confidence intervals for the optimal threshold under a logistic distribution assumption and accounting for the uncertainty of the optimal threshold in the confidence intervals of sensitivity and specificity at this point. Also, a non-parametric analogue has not been investigated so far.

**Simulation study** The simulation study showed that with increasing heterogeneity, the quality of the estimates deteriorates. Generally, reasonable results of the new approach can only be expected for the heterogeneity levels ‘no’ and ‘moderate’. However, since the distribution estimates for almost all data examples have been convincing, we assume that in practice heterogeneity is mostly moderate. Martínez-Camblor [13] in his simulation study considered only levels of heterogeneity smaller than the ’moderate’ heterogeneity level used here. Bias and MSE of the estimates decreased with a increasing number of thresholds per study.

For data with maximally moderate heterogeneity the linear mixed models allowing for different fixed slopes (denoted with *) are to be preferred. They led to smaller bias and MSE in scenarios where the standard deviations were different and to an equivalent bias and MSE in scenarios where the standard deviations were the same.

In most circumstances the bias of sensitivity and specificity was the smallest for the most complex models examined, the CIDS and *CIDS model (common random intercept and different random slope). On the other hand, we observed that the more complex the mixed effects model was, the more convergence problems occurred in the lmer() function.

Unfortunately, the coverage of the estimates of the distribution parameters as well as of sensitivity and specificity was by no means satisfying. This may be due to the existing bias, but more probably to incorrect confidence intervals. For the confidence intervals we assumed the parameters to be approximately normally distributed, but possibly the normal quantiles led to confidence intervals that were too narrow. We also note that the estimates of the ‘true’ sensitivity and specificity depend on how well the distributions and their point of intersection could be estimated. Further, the two-stage model we employed did not account for the uncertainty of estimating sensitivities and specificities at the first level.

Finally, a possible reason for the poor coverage is that we used the standard errors of the fixed effects part of the parameters for estimating the standard errors of the regressions. Methods for integrating the random effects variance into the estimation of confidence intervals, if possible in a one-stage framework, have still to be developed.

Our simulation study was not designed to compare our approach to competing methods. Extensive simulations comparing different methods should be performed in the future.

## Conclusions

Although our new approach can still be improved in some aspects, it accounts for the heterogeneity of the studies and the bivariate character of the data and includes multiple thresholds of studies, possibly differing in number. We proposed a total of 16 linear mixed models which differ in their fixed and random effects structure for estimation of the distribution functions. For model selection, we only considered the models allowing for differing fixed slopes, as they led to better results in the simulation study and applied the REML criterion. However, we would prefer to select the model of choice according to a selection criterion in one step.

Our approach is feasible if all studies used equal measurement methods and if most studies provide information of more than one threshold. Then we may benefit from its advantage that both, an SROC curve and an optimal threshold, can be determined. This is the setting for which we recommend the new approach.

## Abbreviations

AIC, Akaike information criterion; cAIC, conditional Akaike information criterion; cdf, cumulative distribution function; CI, common random intercept; CICS, common random intercept and common slope; CS, common random slope; CIDS, common random intercept and different random slopes; DI, different random intercepts; DICS, different random intercepts and common random slope; DIDS, different random intercepts and different random slopes; DS, different random slopes; DTA, diagnostic test accuracy; FN, false negative; FP, false positive; MSE, mean squared error; REML, restricted maximum likelihood; ROC, receiver operating characteristic; SD, standard deviation; Se, sensitivity; Sp, specificity; SROC, summary receiver operating characteristic; TN, true negative; TP, true positive

## Additional files

Additional file 1 R code for the main function and auxiliary functions for the described method. (TXT 43 kb)

Additional file 2 R code for application of the method to the example data. (TXT 3 kb)

Additional file 3 First example data set in txt format. (TXT 1 kb)

Additional file 4 Second example data set in txt format. (TXT 1 kb)
